# Bovine herpesvirus glycoprotein D: a review of its structural characteristics and applications in vaccinology

**DOI:** 10.1186/s13567-014-0111-x

**Published:** 2014-10-31

**Authors:** Luana Alves Dummer, Fábio Pereira Leivas Leite, Sylvia van Drunen Littel-van den Hurk

**Affiliations:** Laboratório de Bacteriologia, Núcleo de Biotecnologia, Centro de Desenvolvimento Tecnológico, Universidade Federal de Pelotas, Pelotas, Rio Grande do Sul 96010-900 Brazil; Microbiology and Immunology, College of Medicine, University of Saskatchewan, Saskatoon, Saskatchewan S7N 5E3 Canada; VIDO-Intervac, University of Saskatchewan, Saskatoon, Saskatchewan S7N 5E3 Canada

## Abstract

The viral envelope glycoprotein D from bovine herpesviruses 1 and 5 (BoHV-1 and -5), two important pathogens of cattle, is a major component of the virion and plays a critical role in the pathogenesis of herpesviruses. Glycoprotein D is essential for virus penetration into permissive cells and thus is a major target for virus neutralizing antibodies during infection. In view of its role in the induction of protective immunity, gD has been tested in new vaccine development strategies against both viruses. Subunit, DNA and vectored vaccine candidates have been developed using this glycoprotein as the primary antigen, demonstrating that gD has the capacity to induce robust virus neutralizing antibodies and strong cell-mediated immune responses, as well as protection from clinical symptoms, in target species. This review highlights the structural and functional characteristics of BoHV-1, BoHV-5 and where appropriate, *Human herpesvirus* gD, as well as its role in viral entry and interactions with host cell receptors. Furthermore, the interactions of gD with the host immune system are discussed. Finally, the application of this glycoprotein in new vaccine design is reviewed, taking its structural and functional characteristics into consideration.

## Table of contents

1. Introduction
2. Structure of glycoprotein D (gD)
3. Vaccines designed with gD
  3.1. Subunit vaccines
  3.2. DNA vaccines
  3.3. Vectored vaccines
4. Conclusions
5. Abbreviations
6. Competing interests
7. Authors’ contributions
8. References

## 1. Introduction

Herpesviruses constitute a large and diverse family of enveloped viruses and are composed of three subfamilies, *Alpha-*, *Beta-* and *Gammaherpesvirinae*. The viruses classified in the *Alphaherpesvirinae* subfamily share several characteristics including a rapid reproductive cycle and, at least for three genera of this subfamily, the ability of neuronal invasion and establishment of latency in sensory nerve ganglia (reviewed by Engels and Ackermann in 1996 [1]). Important prototypes of this family comprise human herpesviruses, such as *Human herpesvirus* (HHV)-1 and -2 (known as herpes simplex virus (HSV)-1 and -2), and animal herpesviruses. Among alphaherpesviruses infecting ruminants, the prototype is *Bovine herpesvirus 1* (BoHV-1); however, the closely related BoHV-5 is also of great importance in veterinary medicine [2].

BoHV-1 is a pathogen of cattle associated with two major syndromes, called infectious bovine rhinotracheitis (IBR) and infectious pustular vulvovaginitis (IPV) [1]. It is one of the pathogens involved in the bovine respiratory disease complex (BRD), also referred to as “shipping fever”, which economically affects producers by reducing the average daily weight gain, feed efficiency, and overall performance of calves (reviewed in reference [3]). The severe damage that exposure to BoHV-1 can cause to the respiratory tract creates opportunities for further fatal secondary bacterial infections [4,5].

BoHV-5 infection occurs at the same potential entry sites as BoHV-1, i.e. nasal cavity, eyes, oropharynx and genital tract. The first round of replication usually takes place in the epithelial cells at these entry sites, and then the virus can spread to the neurons [6]. Although BoHV-5 and BoHV-1 are genetically and antigenically related, sharing on average 82% of identity in their amino acid sequences [7], they differ in their neuroinvasion and neurovirulence ability. Neuroinvasion of BoHV-1 usually does not progress beyond the first order neuron located in the trigeminal ganglion, where the latent infection is established, whereas BoHV-5 can infect different regions of the brain causing lethal encephalitis in young animals (reviewed in Zajac et al. [8]).

Vaccination is one of the most cost-effective strategies to prevent and control the clinical signs and transmission of these viruses. Conventional modified live and killed vaccines have been developed against BoHV-1; however, several disadvantages regarding safety and/or efficacy make then unsuitable for vaccination of some targets such as pregnant cows (reviewed in reference [9]). New strategies for vaccine development against both BoHV-1 and BoHV-5 have been focused on the design of marker vaccines, which differentiate infected from vaccinated animals (also known as DIVA vaccines). DIVA vaccines include genetically engineered gene-deleted viruses, for example gE^−^ virus, and subunit or vectored vaccines based on a viral envelope glycoprotein such as gD.

BoHV-1 gE^−^ live marker vaccines have been developed and tested for virulence in calves, demonstrating protection after challenge and reduction in virus shedding without any effect on the immunogenicity of BoHV-1 [10,11]. A gE^−^ live marker vaccine has been used in eradication programs in countries with a high prevalence of BoHV-1 infection (reviewed in reference [12]). Field trials of this vaccine were performed [13] and in 2007, a study performed in three European countries demonstrated reduction in the rate of seroconversion observed in vaccinated animals, suggesting efficiency of the vaccination program in containing viral spread [14].

However, the use of a BoHV-1 gE^−^ live marker vaccine [15] conferred limited protection against encephalitis when animals were challenged with BoHV-5, with reduction in the intensity of the clinical signs after primary infection, but without protection against infection and no effect on nasal virus shedding or the development of encephalitic lesions [16]. Thus, the use of BoHV-1 gE^−^ live marker vaccine where BoHV-5 prevalence is higher may be unsuitable. In 2007, Franco et al. [17] reported the construction of a BoHV-5 gI/gE/US9^−^ and the resulting virus has been tested as an inactivated vaccine in 2010 [18], conferring protection to encephalitis after challenge with a high dose of virulent BoHV-5 and reduction of nasal virus shedding in vaccinated animals.

Although successful use of BoHV-1 gE^−^ live marker vaccines has been reported, a number of issues concerning these vaccines have been identified. Contamination of vaccine batches with bovine viral diarrhea virus (BVDV) type 2 leading to BVDV outbreaks [19], false seronegative vaccine virus carriers in calves immunized while carrying maternal antibodies and latency establishment have been reported [20].

Glycoprotein D (gD), one of the antigens present in the viral envelope, is involved in virus penetration and has been considered the major target in vaccine development against bovine and human herpesviruses, mainly owing to its ability to stimulate both humoral and cell-mediated immune responses in the host (reviewed in reference [21]). Subunit, DNA and vectored vaccines based on this glycoprotein have been generated and evaluated with a variety of adjuvants. This review highlights the role of gD in viral pathogenesis and its application in vaccine strategies against bovine herpesviruses, in context of its structural, functional and immunological properties. In consideration of the molecular homology between these viruses [7] most of this review will describe studies with BoHV-1 and BoHV-5. However, although multiple outbreaks of infection by BoHV-5 have been described [22-24] and BoHV-5 infection leads to more serious disease, there are fewer reports on vaccine development strategies against BoHV-5 than against BoHV-1 [18,25]. Where appropriate, studies with gD from other alphaherpesviruses will be described to illustrate the recent progress with gD-based vaccines in general.

## 2. Structure of glycoprotein D (gD)

The complex virion structure of the *Herpesviridae* is common among the viruses of this family. The virion consists of a large double-stranded DNA genome packaged into an icosahedral capsid, which, in turn, is surrounded by a layer of proteins called tegument and an envelope composed of a large number of glycoproteins embedded in a lipid bilayer (reviewed in reference [26]).

The entry of alphaherpesviruses into permissive cells is a complex and not yet fully understood process. These viruses infect cells as free particles and then can spread to adjacent cells through cell contact. Two mechanisms have been proposed for this entry: viral envelope fusion and an endocytic pathway (reviewed in [27,28]). However, it is known that among the 12 identified envelope glycoproteins, at least six (gC, gB, gD, gH, gK and gL) participate in viral attachment and entry. Binding of the alphaherpesvirus to the host cell is attributed to the reversible attachment through gC and/or gB to cell surface heparan sulfate proteoglycans. A detailed description of this interaction has been provided in review [29]. Indeed, the infectivity of alphaherpesviruses is increased when gC is attached to its receptor, but gC null mutants can also infect the cell [30], suggesting that this attachment alone is not sufficient for virus penetration and that more events are required for cell entry. Fusion of the viral envelope with the plasma membrane of the cell requires the other four glycoproteins (gD, gB and the gH-gL complex), with the binding of gD to one of its cell surface receptors being an essential event for herpesvirus entry, targeting a series of interactions between gB and gH-gL that occurs concurrently with fusion [31]. Another component of the fusion mechanism is gK, which also interacts with gB by binding to its amino terminus and modifying the ability of this glycoprotein to mediate “viral-envelope-to-cellular-membrane fusion” [32]. Although deletion of the gene coding for this protein is not lethal for replication in cell culture, titers were reduced indicating that virions defective in gK have an impaired entry process [33].

Glycoprotein D of both BoHV-1 and BoHV-5 consists of approximately 417 aa, with 79.9% amino acid (aa) identity [34], N- and O-linked oligosaccharides, and a molecular weight of approximately 71 kDa [35]. It is a type I membrane glycoprotein, with a signal sequence and a cleavage site located between aa 18 and 19 in BoHV-1 gD and between aa 19 and 20 in BoHV-5 gD, based on the position, length, relative hydrophobicity and consensus cleavage site characteristic of a signal sequence [36]. This signal sequence is cleaved to yield a mature protein of 399 aa. The amino-terminal portion of gD comprises the extracellular domain, while its carboxy-terminus consists of a hydrophobic transmembrane anchor sequence and a cytoplasmic tail of approximately 28 aa in BoHV-1 gD and 35 aa in BoHV-5 gD [35,37].

**Figure 1:**
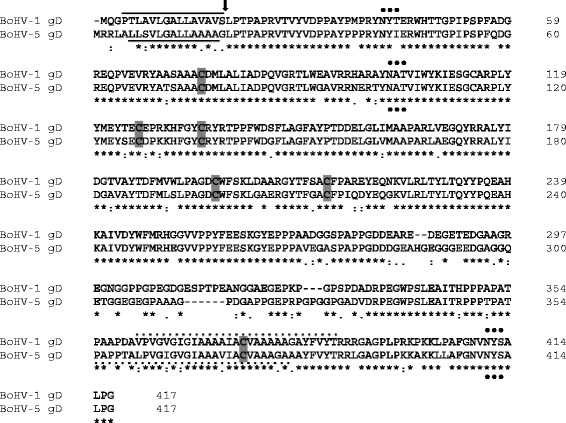
**Comparison of the predicted amino acid sequence of gD from BoHV-1 and BoHV-5.** BoHV-1 gD [GenBank:CAA80604.1] [39] and BoHV-5 gD [GenBank:AAA67359.1] [36] were aligned with Clustal Omega v. 1.2.1 [40]. Numbers are shown on the right, starting at the first methionine. Fully conserved residues are indicated with an asterisk (*); conservation between groups of strongly similar properties are indicated with a colon (:); conservation between groups of weakly similar properties are indicated with a period (.); conserved cysteine residues are indicated in a grey box; potential N-linked glycosylation sites are indicated with (•). Solid and dashed lines show presumed signal sequences and transmembrane anchor sequences, respectively. An arrow indicates the position of the BoHV-1-gD signal peptide cleavage site (aa 18 and 19) [35].

The nucleotide sequence of this glycoprotein gene has a GC nucleotide content of 70% [35], similar to *Suid herpesvirus 1* (SuHV-1) (75%) and HHV-1 (65%) gD homologues [38]. The BoHV-1 and -5 gD amino acid sequence demonstrates conservation of 6 cysteine residues in its ectodomain, suggesting that these may be disulfide bond-linked, which probably plays a role in maintaining the proper 3D-structure and its function [35]. Alignment of both gDs showed that the amino-terminal two-thirds (aa 1-282) of BoHV-1 [35,39] and BoHV-5 [36] gD are relatively well conserved (Figure 1). However, the difference between gD from these viruses maps to a glycine-rich stretch located in the molecule’s C-terminal part of the ectodomain (aa 280 and 330), in close vicinity to the transmembrane region [34,36]. This region is characterized by a major hydrophilic peak in the case of BoHV-1 gD and may be important for interactions of the protein with other molecules of the virus, host cell or both through ionic interactions [37]. This same region in BoHV-5 gD exhibits several mismatches with the BoHV-1 gD sequence, with the presence of a series of negatively charged residues from aa 281 to 295 (280 to 292 of BoHV-1 gD), which results in a series of hydrophilic peaks compared to one broad peak for BoHV-1 gD [36].

Four antigenic domains have been described for BoHV-1 gD [41], and five epitopes, three interrelated and two independent, have been reported as targets of neutralizing antibodies [42]. Monoclonal antibodies developed to epitopes located between amino acids 52-116 (MAb 3402) and 165-216 (MAb R54) do not recognize BoHV-5 gD, suggesting that these epitopes are located in the corresponding altered amino acids in BoHV-5 gD [36].

The most well characterized homologue is HHV-1 gD, the ectodomain of which is formed by a core with a variable-type immunoglobulin fold (IgV) wrapped by a N-terminal extension and a C-terminal proline-rich extension [43]. With respect to its glycosylation, differences are observed in the N-linked oligosaccharide (N-CHO) distribution for gD. HHV-1 and BoHV-1 gD possess three N-CHO sites (amino acids position 41, 102 and 411 of BoHV-1 gD), while BoHV-5 gD has only two potential sites in its sequence, one being located in its cytoplasmic tail (amino acids 102 and 411 of BoHV-5 gD) [36]. The importance of the N-CHO sites in gD is attributed to the influence of glycosylation on the protein structure and antigenic properties rather than to direct interaction with cellular receptors [44].

Enzymatic deglycosylation of gD from BoHV-1 suggests that the addition of carbohydrates could mask epitopes involved in T cell recognition. This was observed after injection of native or deglycosylated forms of gD in rabbits and measurement of the delayed-type hypersensitivity (DTH) response where a stronger DTH reaction in deglycosylated gD-vaccinated rabbits was observed. In contrast, the total antibody response to gD after carbohydrate removal was lower than the response observed for native gD-vaccinated animals; however, the neutralizing antibody response and the ability of the antibodies to mediate cell lysis were not significantly reduced, indicating that most functional epitopes on this glycoprotein are carbohydrate-independent [44].

To date, three unrelated molecules have been shown to be gD cell surface receptors: nectin-1, herpesvirus entry mediator (HVEM) and heparan sulfate modified by 3-O-sulfotransferases, at least for HHV-1. Nectin-1 (also known as HveC and Prr1) is the main receptor of gD on epithelial and neuronal cells [45] and can mediate entry of HHV-1 and -2 as well as SuHV-1 (Pseudorabies virus or PRV), BoHV-1 and BoHV-5 [46]. However, cells expressing heparin sulfate-modified 3-O-sulfotransferases or the HVEM receptor are not susceptible to BoHV-1 (reviewed in 2000 by Spear et al. [47]).

Nectins are homophilic cell adhesion calcium-independent molecules from the immunoglobulin (Ig)-like superfamily that accumulate at adherent junctions of epithelia, at synapses and puncta adherentia of neurons [48,49]. All of the nectins have an ectodomain comprising three Ig-like domains (V-C1-C2), a transmembrane and a cytoplasmic tail region. The gD-binding region of nectin-1 is localized at the V-like domain [50]. Studies performed with soluble forms of gD from HHV-1, HHV-2 and SuHV-1 showed efficient binding to truncated forms of nectin-1 retaining only the V-like domain; this binding was blocked by monoclonal antibodies specific for epitopes located in this domain [51,52]. However, a study performed with chimeras that combine the V-like domain of nectin-1α with C-like domains from nectin-2α demonstrates that the V-like domain is required for full entry activity of BoHV-1, HHV-1 and -2, and SuHV-1, only when this domain is linked to two C-domains [53]. Despite the ability of gD to bind to the V-like domain, attachment does not result in viral entry, suggesting that binding of gD induces conformational changes in both gD and the receptor. This study also suggests that, based on competitive interactions, the regions on nectin-1 to which gD from different alphaherpesviruses binds, are overlapping but not necessarily identical [53].

The function of nectins as adhesion molecules that are mostly located at cell junctions suggests a key role in cell-to-cell spread of alphaherpesviruses to neurons of the peripheral and central nervous systems, as well as in the epithelium [45,54]. Binding of gD to nectin-1 during later phases of HHV infection correlates with down-regulation of nectin-1 in cells susceptible to HHV endocytosis. Co-culture of nectin-1-null cells expressing gD with target cells that express nectin-1 indicates that the trans-interaction of gD with nectin-1 at contacts between the two cell populations led to down-regulation of nectin-1, consisting of internalization of the receptor from the target cell surface followed by low-pH-dependent degradation suggesting a ligand-induced endocytosis. The cell-bound gD or gD from egressing virions could then mediate internalization of nectin-1 directing HHV to an endocytic pathway during entry [45].

Madin Darby bovine kidney (MDBK) cells expressing gD from BoHV-1 resist infection by heterologous virus, such as HHV-1 or SuHV-1, a phenomenon termed interference, which occurs at the level of penetration into cells [55]. However, these cells are still susceptible to BoHV-5, demonstrating an absence of interference, even though the presence of gD inhibited increase in plaque size. This observation suggests that BoHV-5 may use different receptors for entry or that different regions of gD from BoHV-5 may be critical for entry [56]. Indeed, a study showed that BoHV-5 gD can interact with human nectins 1 to 4. Also, a cell type known as J1.1-2 cells, which does not express any form of nectin and is resistant to HHV and BoHV-1 infection, is susceptible to BoHV-5 infection, reinforcing the idea that BoHV-5 gD can interact with a different range of receptors than those used by BoHV-1 [34]. Replacement of BoHV-1 gD by the homologous BoHV-5 gD confers an extended host range to BoHV-1 and increased virulence, which, however, does not affect the brain invasiveness of this recombinant virus [34].

The role of gD in the initial stage of BoHV-1 and -5 infection and its abundance in the viral envelope makes it a target for the host immune system. The host immune responses to these viruses can be divided into innate responses mediated by polymorphonuclear neutrophils (PMN), macrophages, natural-killer cells (NK-cells), natural-killer T cells (NKT-cells) and dendritic cells (DCs), and adaptive responses by B and T lymphocytes, which are responsible for antibody production (which may prevent infection) and cytotoxic T cell (CTL) killing (which, together with humoral responses, aid in recovery). The immunology of BoHV-1 infection was reviewed in 1996 [21], and very few studies have been performed to elucidate the immune response induced by BoHV-5 infection. However, due to the similarities between both viruses, it is reasonable to expect that most of what was discovered for BoHV-1 may apply to BoHV-5 (reviewed in [8]). The first step in an immune response to a virus infection is to avoid the interaction between virus and permissive cells; however, during primary infection, antibodies are not available to interfere with these interactions. Thus, the first response of the immune system against a BoHV-1 infection will consist of nonspecific inflammatory and cell-mediated reactions, gD and gB being primary targets for NK-cells [57]. The innate responses lead to adaptive immune responses later during infection. Glycoproteins gC and gD are targets for CD8+ CTLs in bovines, and gD possesses specific epitopes which stimulate CD4+ T-lymphocytes [58]. Antibody responses, as mentioned, do not prevent cell-to-cell spread and become detectable when recovery from primary infection is underway. However, during secondary exposure they play a pivotal role in preventing infection, since they are critical in neutralizing extracellular virus and may prevent spread to neighboring animals. Non-neutralizing antibodies may also contribute together with PMNs, which can cause lysis of BoHV-1 infected cells via antibody-dependent cell cytotoxicity (ADCC) [59].

Thus, it becomes clear that the major glycoproteins are not only involved in inducing antibody that may protect the animal from disease, but also in stimulating cell-mediated responses, as they are also targets for CTLs and ADCC. For these reasons, it is important that new vaccine strategies against BoHV-1 and -5 focus on or include the major glycoproteins, in particular gD (reviewed in [21]).

## 3. Vaccines designed with gD

### 3.1 Subunit vaccines

Subunit vaccines consist of one or more pure or semi-pure antigens. To develop subunit vaccines, it is important to identify the individual components that are involved in protection from the pathogen. This kind of vaccine may be produced by conventional technologies, such as purification of the protein produced by the pathogen. To develop a safe vaccine against BoHV-1, viral envelope glycoproteins, gB, gC and gD, were purified directly from virus-infected cells and retained their antigenic activity, inducing the production of neutralizing antibodies in cattle and protection against challenge with virulent BoHV-1. Although all three glycoproteins induced neutralizing antibodies, titers were higher in the animals immunized with gD, and these animals also had higher ADCC titers [60,61].

While purified native glycoproteins from infected cells retain their antigenic characteristics, this method is generally not cost-effective, so the use of recombinant DNA technology to produce large quantities of proteins for incorporation into vaccines may fulfill the safety and economic requirements. Production of recombinant BoHV-1 gD has been achieved in several expression systems, from prokaryotes to eukaryotes. Prokaryotic systems, although possessing several advantages regarding manipulation, low cost and the possibility to achieve high amounts of protein production, have some disadvantages regarding the expression of viral glycoproteins. As mentioned, the conformational structure of gD relies on its correct three-dimensional folding, which at least partially is dependent on its insertion into the endoplasmic reticulum and on the addition of carbohydrates. Prokaryotic systems do not possess the cell machinery to perform post-translational modifications necessary for proper gD folding. This was confirmed when BoHV-1 gD was expressed in *Escherichia coli.* Despite the higher total antibody levels induced by the *E. coli*-expressed recombinant gD (rgD), only a small portion of those were capable of neutralizing the virus [62].

As a result of the low efficacy of recombinant gD produced in prokaryotes, eukaryotic expression systems were tested, such as yeast, mammalian, plant and insect cells. Yeast, such as the methylotrophic *Pichia pastoris,* was used as expression system for a secreted form of gD from BoHV-1 alone, or in combination with bovine interleukin (IL)-6 as a chimeric protein. Both recombinant proteins induced neutralizing antibodies in mice [63,64]. Furthermore, we expressed the secreted form of gD from BoHV-5 in *P. pastoris* by removing the transmembrane anchor of the native gD [65]. Recombinant BoHV-5 gD when formulated with oil-based adjuvants induced neutralizing antibodies in mice [25] and bovines (Itauá Leston Araújo, 2014 - unpublished observations) after vaccination.

A Tobacco Mosaic Virus (TMV)-based vector (TMV-30B) was used to express a cytoplasmic non-glycosylated form of BoHV-1 gD in plant cells. Mice and cattle immunized with an oil-based gD vaccine formulation developed humoral and cell-mediated immune responses, and seemed to be partially protected from disease after viral challenge; however, no viral neutralization test was performed in this trial [66]. Although yeast, plant and insect cells were able to produce gD with some degree of authenticity, most reports are based on a secreted form of BoHV-1 gD (also called tgD) produced in MDBK cells under the control of an inducible bovine heat shock 70A gene promoter (HSP70 promoter) [67]. High levels of BoHV-1 gD, when constitutively expressed in MDBK cells, proved toxic to cells, stable cell lines being established only when the BoHV-1 gD was placed under the control of an inducible promoter [35] or when expressed at basal levels [68]. This problem was solved by removing the transmembrane anchor, resulting in a secreted version of gD with equal immunogenicity to full-length gD [69,70].

**Figure 2:**
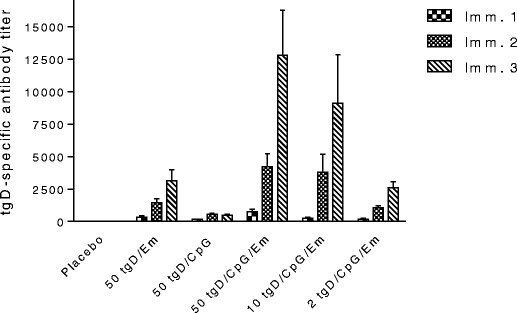
**IgG gD-specific titers in calves vaccinated with different doses and formulations of BoHV-1 tgD.** tgD was formulated with 15% (v/v) of Emulsigen in the absence or presence of 250 μg of CpG ODN. Antibody titers were determined by enzyme-linked immunosorbent assay (ELISA). The procedures were performed in accordance with the standards stipulated by the Canadian Council on Animal Care.

BoHV-1 tgD has been combined with several adjuvants and co-adjuvants and administered by different routes to induce systemic and mucosal immune responses. One of the most effective approaches seems to be the incorporation of CpG-containing oligodeoxynucleotides (ODN) in vaccine formulations with classical adjuvants. Cell-mediated immune responses are critical for protection from several pathogens, and, although all currently licensed vaccines are efficient at inducing antibody responses, only modified live vaccines efficiently induce cell-mediated immunity. However, experimental subunit vaccines when formulated with the appropriate adjuvants may induce cell-mediated immune responses due to cross-presentation. Formulation of tgD with alum and CpG ODN (or even tgD with CpG ODN alone) induced strong neutralizing antibodies and cell-mediated immune responses in calves, resulting in fast recovery from BoHV-1 challenge [71]. The incorporation of CpG ODN in oil-based vaccines against BoHV-1 also resulted in strong Th1-type immune responses, with an increase in production of interferon-gamma (IFN-γ), or balanced immune responses, in contrast to the immune responses induced by the conventional adjuvants alone, which were Th2-biased in mice [72]. On the other hand, with respect to experimental vaccines against BoHV-5, mixed Th1/Th2 immune responses were observed when we vaccinated mice with recombinant BoHV-5 gD formulated only with oil-based adjuvant. This resulted in induction of IFN-γ as well as pro-inflammatory cytokines, such as IL-17 and granulocyte-macrophage colony-stimulating factor (GM-CSF) [25]. More importantly, BoHV-1 tgD formulated with both CpG ODN and Emulsigen elicited a more balanced immune response, and higher levels of virus neutralizing antibodies, compared to BoHV-1 tgD adjuvanted with Emulsigen, as well as full protection from disease after BoHV-1 challenge [73]. Interestingly, the formulation of BoHV-1 tgD with Emulsigen and CpG ODN also induced robust, balanced immune responses in newborn calves, which were almost completely protected from disease after a high-dose BoHV-1 challenge [74]. The addition of CpG ODN to the tgD-Emulsigen formulation allowed us to reduce the antigen dose 5- to 25-fold without decrease in the antibody titers induced in calves (Figure 2); thus, the co-adjuvanting with CpG ODN resulted in antigen sparing which is important for potentially making a gD subunit vaccine commercially viable.

### 3.2 DNA vaccines

The demonstration that direct transfection in vivo with plasmid DNA could be used to express foreign proteins and thereby induce both humoral and cell-mediated immune responses in a variety of murine and primate disease models has provided a new opportunity to develop non-replicating vaccines that induce balanced immune responses. Immunization by direct transfection in vivo with plasmid DNA encoding protective antigens is an efficient means of eliciting CD8+ CTLs, which means that protein antigens produced by DNA vaccination gain access to pathways of antigen presentation via Class I MHC molecules, a system that is most frequently utilized by live attenuated virus vaccines (reviewed by Donnelly et al. [75] and Gurunathan et al. [76]). DNA vaccines attracted attention owing to the immune responses generated, which closely resemble those induced by natural infection, due to endogenous production of viral proteins and glycoproteins [77].

The earliest DNA vaccine developed against BoHV-1 was engineered with a pRSV plasmid, which contains transcriptional control sequences from rous sarcoma virus, and is functional in a wide range of animal cell types [78]. Three glycoproteins were tested individually in mice, and neutralization titers specific for gD after intramuscular injection were higher than those obtained for gB or gC [79]. However, intramuscular immunization of cattle with a gD DNA vaccine resulted in low humoral immune responses. Improved immune responses were achieved by replacing the RSV promoter with the *Human cytomegalovirus* (HCMV) immediate-early promoter/enhancer, expressing the secreted form (tgD) and immunizing intradermally. This approach resulted in balanced and strong immune responses characterized by the induction of tgD-specific antibody titers and high numbers of IFN-γ-secreting cells, sufficient to provide significant protection from clinical signs after BoHV-1 challenge [80]. In contrast, in another study in 2004 [81], a DNA vaccine encoding a truncated form of BoHV-1 gD was tested in calves through three different routes: intramuscular, intradermal and intranasal. Only intramuscular injection resulted in neutralizing antibodies and in early clearance of viral shedding after challenge. However, in mice a plasmid encoding secreted forms of gD and gB and administrated by intranasal route induced high neutralizing antibody titers [82]. Incorporation of multiple copies of CpG motif into a tgD-encoding plasmid also resulted in lymphocyte proliferation and cell-mediated immunity and, following BoHV-1 challenge, high neutralization titers and lower levels of virus shedding, but no difference in clinical symptoms [83]. Plasmid encoding tgD linked to bovine CD154 administered by intradermal immunization was able to bind CD40-expressing DCs present in the skin; however, no enhanced immune response or protection from disease was induced by tgD-CD154, when compared to tgD alone [84]. One of the currently more promising approaches to improve the transfection efficiency of plasmids in vivo, and thus the level of antigen expression, is delivery by electroporation. We demonstrated that by using a TriGridTM Delivery System for intramuscular delivery (Ichor Medical Systems), a bovine viral diarrhea virus (BVDV) vaccine induced balanced immune responses and close to complete protection from clinical disease following challenge with BVDV-2 [85]. As electroporation is being extensively tested in humans and the equipment adapted for easy use, this might be an approach that can be evaluated for delivery of plasmids encoding gD from BoHV-1 and BoHV-5.

### 3.3 Vectored vaccines

Vectored vaccines are also expected to induce balanced immune responses once the cell endogenously produces the protective antigen. One of the main advantages of this kind of vaccine is the potential to deliver the antigen directly to the mucosal surfaces and also, the ability to induce humoral and cell-mediated immune responses.

Due to the ability of bovine adenovirus 3 (BAV-3) to replicate in the respiratory tract of cattle while producing mild or no clinical symptoms, and to grow to high titers in cell culture [86], BAV-3 expressing gD was used to immunize cattle. Intranasal immunization with a replication-competent BAV-3 expressing full-length gD or tgD produced gD-specific immune responses, including IgA in the nasal secretions. However, although animals were partially protected after BoHV-1 challenge, the magnitude of the IgA response was not sufficient to eliminate BoHV-1 shedding [87]. The same pattern of response was observed by immunization of cattle through intratracheal/subcutaneous routes [88].

Human adenovirus 5 (HAdV-5) has also been used as viral vector to deliver BoHV-1 glycoproteins. Replication-defective HAdV-5 expressing gC or gD under the control of the HCMV immediate-early promoter/enhancer or human desmin gene 5′regulatory region (DESM) promoter was used to induce immune responses in a rabbit model. A single intranasal immunization with the HAdV-5 expressing gD alone or in combination with HAdV-5 expressing gC, both under the control of the CMV promoter, elicited neutralizing antibodies, the titers elicited by gD being much higher than those induced by gC. The same was observed after intramuscular administration of HAdV-5 expressing gD under the control of the DESM promoter; however, the neutralizing antibodies were lower than those induced by intranasal immunization [89]. This vector expressing gC and gD induced virus neutralizing antibodies and clinically protected cattle from BoHV-1 challenge after intranasal administration [89]. The same vector construct was further used in combination with glycol chitosan, also demonstrating protection against challenge [90]. No cell-mediated immune responses were evaluated in these trials. A summary of the principal results of vaccines formulated with gD from BoHV-1 and BoHV-5 is presented in Table 1.

**Table 1:** **Principal results obtained with BoHV-1 and BoHV-5 gD vaccines**

**Vaccine**	**Virus**	**Expression**	**Adjuvant**	**Route**	**Immune Resp.**		**Protection**	**Ref.**
		**system**			**Humoral**	**Cellular**		
Subunit	BoHV-1	Native gD	Avridine	i.m.	++++	n/a	xxxx	[60,61]
	BoHV-1	*E. coli*	Avridine	i.m.	++	n/a	xx	[62]
	BoHV-1	*P. pastoris*	Oil	i.m.	++++	++	n/a	[63,64]
	BoHV-5	*P. pastoris*	Oil	i.m.	++++	++	n/a	[25]
	BoHV-1	MDBK	Oil	i.m.	++++	++	xx	[69,70]
	BoHV-1	MDBK	CpG; Alum	i.m.	++++	++++	xxxx	[71]
	BoHV-1	MDBK	CpG; Oil	s.c.	++++	++++	xxxx	[72]
	BoHV-1	Plant cells^a^	Oil	i.p., s.c, i.m.	n/a	n/a	xx	[66]
DNA	BoHV-1	pRSV	n/a	i.m.	++++	n/a	xx	[79]
	BoHV-1	HCMV	n/a	i.d.	++++	++++	xxxx	[80]
	BoHV-1	HCMV	n/a	i.m.	++++	++++	xxxx	[82]
	BoHV-1	HCMV	CpG	i.d.	++	++++	xx	[83]
Vectored	BoHV-1	BAV-3	n/a	i.n	++	++++	xx	[87,88]
	BoHV-1	HAd5	n/a	i.n	++	n/a	xxxx	[89,90]

a = achieved with a Tobacco Mosaic Virus (TMV)-based vector.

++ = Weak neutralizing antibodies or cell-mediate response.

++++ = Strong neutralizing antibodies or cell-mediate response.

n/a = Data not available.

xx = Partial protection.

xxxx = Total protection.

The gD from another alphaherpesvirus, the *Caprine herpesvirus 1* (CpHV-1), has recently been expressed in the genome of a gammaherpesvirus, *Bovine herpesvirus 4* (BoHV-4). Subcutaneous administration of the engineered BoHV-4 carrying the CpHV-1 gD gene protected animals from clinical signs and reduced viral shedding after challenge [91]. Although this reinforces the success of the gD vaccines in stimulating the host immune system, protecting from clinical signs and diminishing viral shedding, these results have not always been achieved with other alphaherpesviruses, as was observed for HHV-1 and HHV-2.

When gD of both HHV-1 and HHV-2 as vaccine antigen was tested in animal models, this glycoprotein indeed induced immune responses capable of mediating clinical protection and reduction of viral shedding [92,93]. In a prophylactic trial with guinea pigs HHV-1 gD expressed in mammalian cells induced neutralizing antibodies to both HHV-1 and HHV-2, protecting from clinical symptoms after challenge by intravaginal infection with HHV-2 [92]. However, trials performed in humans did not demonstrate similar results (reviewed in 2003 by Koelle and Corey [94]). The HHV-2 gD combined with gB and the adjuvant MF59, although capable of eliciting HHV-2-specific neutralizing antibodies, only produced partial and transient clinical protection from HHV-2 [95]. In trials using HHV-2 gD combined with alum and 3′-O’deacylated-monophosphoryl lipid A (MPL) the vaccine was well tolerated and protected women with no preexisting HHV antibodies from disease, but failed to protect men and did not add to the protection provided by previous HHV-1 infection in women [96,97].

In 2012, these trials were repeated indicating that the vaccine was not efficacious with regard to protection against genital disease caused by either HHV-1 and HHV-2 after two administrations, whereas three doses were associated with efficacy against HHV-1, but not HHV-2 [98]. Subsequent studies correlated the administration of the vaccine with protection from HHV-1 disease, attributing these results to the homology between the region of HHV-2 gD used in the vaccine with the corresponding region of HHV-1 gD. Although the vaccine stimulated neutralizing antibodies, those failed to protect from clinical symptoms caused by HHV-2, which, as suggested by the authors, may indicate a need to develop new vaccine strategies to stimulate higher antibody titers against HHV-2 [99].

Recent trials with HHV-2 gD also implicate the need to stimulate proper cell-mediated immunity for protection from disease caused by HHV. Combination of HHV-2 gD with immediate-early proteins, such as ICP27 or ICP4 that stimulate stronger T cell immunity, enhanced the level of clinical protection when compared to gD alone, when tested in mice or guinea pigs followed by challenge with HHV-2 [93,100].

## 4. Conclusions

Glycoprotein D homologues of alphaherpesviruses play a major role in viral interactions with permissive cells and are very immunogenic. For these reasons, gD has been employed in vaccine candidates over the last decades and may still be applied as long as new immunization strategies are developed. Knowing its characteristics and what has been achieved so far with this glycoprotein is important to develop further approaches to minimize economic losses caused by BoHV-1 and BoHV-5 in cattle worldwide. Subunit gD vaccines have been shown to be very efficacious, but have the drawback of being expensive for use in cattle. This may be overcome by formulation with effective adjuvants, in particular combinations like CpG ODN and Emulsigen, such that the antigen dose can be reduced making the vaccine more affordable. The reports presented in the literature so far indicate that, although DNA vaccines encoding gD are suitable for the induction of cell-mediated immune responses, improvements are still needed to achieve a balanced response where both cell-mediated and humoral immune responses are present. This means that better delivery systems need to be developed to improve the transfection efficiency in vivo and level of antigen expression in the vaccinated animals. Vectored gD vaccines are particularly promising for induction of mucosal immunity, but also need to be improved. Virus neutralizing antibodies constitute one of the most important correlates of clinical protection for BoHV-1 and BoHV-5. This suggests that at this stage a gD subunit vaccine formulated with an adjuvant that promotes a balanced immune response best fulfills the requirements to induce strong humoral and cell-mediated immunity.

## Abbreviations

aa: Amino acid
ADCC: Antibody-dependent cell cytotoxicity
BAV-3: Bovine adenovirus 3
BoHV-1: *Bovine herpesvirus 1*
BoHV-4: *Bovine herpesvirus 4*
BoHV-5: *Bovine herpesvirus 5*
BRD: Bovine respiratory disease complex
BVDV: *Bovine viral diarrhea virus*
CD154: Cluster of Differentiation 154
CD4+: Cluster of Differentiation 4 positive
CD40: Cluster of Differentiation 40
CD8+: Cluster of Differentiation 8 positive
CMV: Cytomegalovirus
CpG: Cytosine phosphodiester guanine
CpHV-1: *Caprine herpesvirus 1*
CTL: Cytotoxic T lymphocyte
DC: Dendritic cells
DESM: Human desmin gene 5′ regulatory region
DNA: Deoxyribonucleic acid
DTH: Delayed-type hypersensitivity
gB: Glycoprotein B
gC: Glycoprotein C
gD: Glycoprotein D
gH: Glycoprotein H
gL: Glycoprotein L
GM-CSF: Granulocyte-macrophage colony-stimulating factor
HAdV-5: human adenovirus 5
HCMV: *Human cytomegalovirus*
HHV-1: *Human herpesvirus 1*
HHV-2: *Human herpesvirus 2*
HSP70: Heat shock protein 70
HSV-1: Herpes simplex virus 1
HSV-2: Herpes simplex virus 2
HveC: Herpesvirus entry mediator C
HVEM: Herpesvirus entry mediator
IBR: Infectious bovine rhinotracheitis
ICP: Infected cell protein
ICP27: Immediate-early protein 27
ICP4: Immediate-early protein 4
i.d.: intradermal
IFN-γ: Interferon-gamma
Ig: Immunoglobulin
IgA: Immunoglobulin A
IgV: Variable-type immunoglobulin
IL-17: Interleukin 17
IL-6: Interleukin 6
i.m.: intramuscular
i.n.: intranasal
IPV: Infectious pustular vulvovaginitis
kDa: kilo-Dalton
MAb: Monoclonal antibody
MDBK: Madin Darby bovine kidney cells
MHC: Major histocompatibility complex
MPL: 3′-O’deacylated-monophosphoryl lipid A
N-CHO: N-linked oligosaccharide
NK-cells: Natural-killer cells
NKT-cells: Natural-killer T cells
ODN: Oligodeoxynucleotides
PMN: Polymorphonuclear neutrophils
Prr1: Poliovirus receptor-related-1
pRSV: Rous sarcoma virus plasmid
PRV: Pseudorabies virus
rgD: Recombinant gD
s.c.: subcutaneous
SuHV-1: *Suid herpesvirus 1*
tgD: Secreted or truncated form of gD
Th1: Type 1 helper T cell
Th2: Type 2 helper T cell
TMV: *Tobacco mosaic virus*
